# Diabetes and Associated Cardiovascular Complications in American Indians/Alaskan Natives: A Review of Risks and Prevention Strategies

**DOI:** 10.1155/2018/2742565

**Published:** 2018-09-13

**Authors:** Anil Poudel, Joseph Yi Zhou, Darren Story, Lixin Li

**Affiliations:** ^1^Department of Physician Assistant, College of Health Professions, Central Michigan University, MI 48859, USA; ^2^College of Medicine, Central Michigan University, MI 48859, USA; ^3^Program in Neuroscience, Central Michigan University, MI 48859, USA

## Abstract

Diabetes mellitus (DM) is the seventh leading cause of death in the United States and the leading cause of death in the U.S. American Indian/Alaskan Natives (AI/ANs), who comprise only 2% of the total population. The AI/AN population has a high prevalence of DM in adults aged 20 years or older and is developing DM at a younger age than the general U.S. population. DM is a major risk factor for cardiovascular disease (CVD), and mortality from CVD is higher in AI/ANs than the general population, as is the prevalence of stroke and 1-year poststroke mortality for both genders when compared to non-Hispanic whites. A genome-wide scan found a number of chromosome linkages in the AI/AN population that suggest that genetic factors may contribute to their high risk of DM and CVD. Importantly, studies also suggest that in addition to race/ethnicity, cultural norms and historic conditions play important roles in the prevalence of DM and CVD in this population. Therefore, multiple factors should be taken into consideration when establishing prevention programs to decrease the prevalence of obesity, diabetes, and CVD incidence among adults and children in the AI/AN population. Prevention programs should focus on behavioral risk factors and lifestyle changes like encouraging smoking cessation, healthy diet, and increased physical activity while taking into consideration cultural, economic, and geographic factors.

## 1. Introduction

Diabetes mellitus (DM) is the seventh leading cause of death in the United States. According to the 2017 National Diabetes Statistic report, 9.4% (30.3 million people) of the U.S. population are affected by diabetes and 33% of U.S. adults aged 18 years and older have prediabetes [1]. DM is a major risk factor for cardiovascular disease (CVD), with DM patients being two to four times more likely to develop CVD than the general population [2]. In addition, both DM and insulin resistance are known to increase the risk of developing heart failure independent of age, gender, coronary artery disease (CAD), or hypertension and are associated with worse clinical outcomes [3]. Furthermore, CVD is the leading cause of morbidity and mortality in those diagnosed with DM, and the mortality rate from heart failure is almost doubled in patients who have diabetes when compared to their normal glycemic counterparts [4].

Although diabetes affects all races, the Centers for Disease Control and Prevention (CDC) report on DM indicates that the American Indian/Alaskan Native (AI/AN) populations, who comprise only 2% of the U.S. population, have a higher prevalence of DM in those aged 20 years or older, with a prevalence more than two times that of the general population [5]. DM is the fourth leading cause of death in the AI/AN population [3]. Additionally, AI/AN youths are disproportionately affected by obesity, a major risk factor for DM, compared to other ethnic groups in the United States, with 40 to 50% of children in many AI/AN communities being reported as overweight or obese [6, 7]. As is the case in the U.S. population in general, CVD is also the leading cause of death in the AI/AN population [8, 9]. However, the incidence of CVD in this population shows considerable variability, ranging from 15 to 28 per 1000 among AI/AN men and only 9 to 15 per 1000 in AI/AN women ages 45–74 [10–12], and the factors for this discrepancy remain unclear.

To date, there have been very few comprehensive reviews of the literature on the prevalence of DM and DM-related CVD complications among the AI/AN population. In the current review, we summarize findings on the prevalence of DM and CVD, analyze the risk factors contributing to the high prevalence of these diseases in the AI/AN population, discuss strategies for disease prevention, and address the existing health disparities.

## 2. The Prevalence of DM Is High in the AI/AN Population and Is Associated with Younger Age of Onset and Higher Risk of Developing CVD

### 2.1. The Prevalence of DM Is High in AI/AN Populations and Increasing, with a Trend toward Younger Age of Onset

The prevalence of DM is consistently high in AI/AN populations of all ages and genders [13–17] and is almost three times that of U.S. non-Hispanic whites when age is adjusted [10]. In addition, an alarming increase in DM prevalence among AI/AN was revealed in the Behavioral Risk Factor Surveillance System (BRFSS surveys) which found an increase of 29% in the incidence of DM between 1990 and 1997 in the AI/AN population [18]. A similar increase in DM prevalence among AI/ANs was also observed by the BRFSS from 2000 to 2006, as well as a similarly increasing trend compared to non-Hispanic whites from 2004 to 2008 observed in data from the National Health Interview Survey (NHIS) [10]. However, the increased rate of DM prevalence in the AI/AN population has large discrepancies from region to region, ranging from 16% in the Northern Plains to as high as 76% in the Alaskan region based on the Indian Health Services (IHS) national outpatient database [10]. Specifically, the prevalence of DM increased by 47% in AI/AN adults 20–24 years old and by 50% for those 25–34 years of age [19]. The prevalence of DM in older adults (>55 years) was also higher compared to that of non-Hispanic whites (21.9% and 13.0%, respectively) based on BRFSS aggregated data from 2001 to 2004 [20]. Importantly, an association with younger age of individuals who develop DM among AI/AN populations compared to the U.S. diabetic population has been identified [10], with the number of children, adolescents, and young adults diagnosed with diabetes increasing by 71% based on the IHS national outpatient database from 1990 to 1998 [19]. AI/AN women had the highest prevalence of DM among the U.S. population [21, 22], with 5.4% of AI/AN women between 18 and 44 years of age affected (BRFSS data 2005–2007) [10], compared to only 2.2% of non-Hispanic whites [23]. Data from the Well-Integrated Screening and Evaluation for Women Across the Nation (WISEWOMAN) study confirmed this finding [24]. It should be mentioned that although the advantage of national survey data is admitted, these self-reported data probably have significant biases which should be taken into consideration when making comparisons between populations.

Regional variations in DM control and morbidity among AI/AN populations have also been noted. For instance, 27% of DM patients under age 45 have plasma hemoglobin A1c levels greater than 9.0 in the Alaskan region, versus 56% in the Southwest AI population [25]. Variations in DM prevalence and diabetes control among older individuals were also obvious in these regions [25]. AI adults with DM were also more likely to present with diagnoses of hypertension, renal failure, lower extremity amputations, neuropathy, mental health disorders, and substance abuse with comorbid liver disease in the Phoenix area compared to other U.S. adults with DM [26].

### 2.2. AI/AN Populations Have a High Risk of Developing CVD and an Associated Increase in CVD Mortality

Heart disease is the leading cause of death among AI/AN populations, with CVD resulting in 1813 deaths in 2009 [27] and 3288 in 2014 [28]. Self-reported heart disease, defined as the existence of coronary heart disease (CHD), angina, heart attack, or other heart conditions or diseases in the AI/AN population, is higher than in Non-Hispanic whites (14.7% compared to 12.2%, respectively) [29]. These disparities in the prevalence of CVD among AI/AN populations were also seen in a number of other studies [30–34].

The Strong Heart Study (SHS), supported by the National Heart, Lung, and Blood Institute (NHLBI), is the largest epidemiological study yet conducted to have examined cardiovascular risk factors in the AI population [35]. The SHS revealed that cardiovascular mortality was higher in AI populations compared to the general U.S. population [13, 36]. Similarly, registration data with the National Death Index indicated that the mortality rate from heart disease was significantly higher among AI/ANs than whites aged 35 years and older from 1990 to 2009 [31, 37]. The incidence of congestive heart failure (CHF) among AI/AN men, however, was higher than AI/AN women and was associated with a worse prognosis. In agreement with these findings, further analysis of echocardiograms from participants in the SHS aged 45 to 74 years old revealed that AI/AN women have much better left ventricular (LV) contractility and greater LV myocardial and chamber function than AI/AN men [38–42].

### 2.3. Type 2 Diabetes Is Associated with a High Prevalence of CVD Complications in the AI/AN Population

It has been well documented that type 2 diabetes (T2D) patients have a greater risk for cardiovascular morbidity and mortality in the general population [43]. Recent research suggests that cardiac death, rate of adverse cardiovascular outcomes such as readmission for acute coronary syndrome (ACS), and heart failure were higher in patients with DM [44]. DM patients with nonobstructive coronary artery stenosis (NOCS; 20%–49% luminal stenosis) after a first non-ST-elevation myocardial infarction (NSTEMI) had a worse prognosis. However, treatment with a GLP-1 analogue was proven to result in a significant improvement on clinical outcomes [44]. The association between autonomic dysfunction and silent atrial fibrillation (AF) was also observed in T2D patients younger than 60 years in one study [45]. Furthermore, several studies suggest that DM has direct adverse effects on cardiac structure and function independent of the severity of CAD but is highly associated with increased LV wall mass, LV torsion, and decreased myocardial perfusion [46]. This correlation was also observed in the AI/AN population [42, 47–51]. A multinational study comparing the vascular disease incidence in younger diabetic patients who were diagnosed with DM before the age of 30 revealed that AI men had higher rates of renal failure and lower extremity amputation than other ethnic groups [52]. The same study also reported higher incidences of retinopathy, clinical proteinuria, and albuminuria in that population.

It has been shown that systemic hypertension (SH) increases the risk of CVD in diabetic patients [53]. DM and SH were both significantly associated with adverse effects on LV wall structure and function in the AI population after adjusting for age, gender, BMI, and heart rate [54]. Furthermore, the impact of DM and SH, when combined, was associated with more severely abnormal LV relaxation, a greater degree of LV hypertrophy, myocardial dysfunction, and arterial stiffness than either condition alone in AI/AN populations [54].

### 2.4. AI/AN Populations Have a Higher Risk of Stroke and Poststroke Mortality

CHD and stroke share several risk factors such as hypertension, smoking, diabetes, physical inactivity, and obesity. Patients with CAD have an increased risk of developing stroke. Recent studies suggest that subclinical episodes of atrial fibrillation (AF) occurred frequently in T2D patients and are associated with an increased risk of silent cerebral infarct (SCI) and the development of stroke in T2D patients who are younger than 60 years of age [55]. Additionally, 9% of the diabetic patients developed stroke even if they were treated with antiplatelet medications [55]. Stroke is the seventh leading cause of death among AI/ANs, and the mortality rate from stroke is also higher than that of whites (29.5 per 100,000 for AI/ANs compared to 24.0 per 100,000 for whites) [56, 57]. Furthermore, the one-year poststroke mortality rate in AI women (33.1%) and AI men (31%) is higher than in the general population for both genders (24% for women and 21% for men, respectively) [58, 59].

When compared to the non-Hispanic white population, the prevalence of stroke in AI/ANs was higher (2.4% and 4.7%, respectively), with a concomitantly higher risk ratios than whites for all three stroke subtypes [36]. Those SHS participants who had not had a stroke at baseline (from 1989 to 1992) were followed until the end of 2004, and the incidence of stroke was found to be much higher in AI/AN participants [36].

## 3. Genetics, Heritability, and Other Risk Factors Contribute to the Increased Incidence of CVD in the AI/AN Population

### 3.1. Genetic Factors and Heritability Contribute to the High Risk of DM and CVD in the AI/AN Population

Genetic factors and heritability may play an important role in the high prevalence of CVD observed in the AI/AN population. To explore this possibility, SHS initiated a genetic epidemiology study and a full family study to explore the potential impact of genetic factors and heritability on the prevalence of these disorders in the AI/AN population [60, 61]. The SHS pilot family study recruited 10 large families from each of three field centers in addition to the original cohort. Subsequently, approximately 30 families (10 per field center) with more than 900 family members participated in the study. The CVD risk factors and heritability were also investigated. Localization of genes that contribute to CVD risk was conducted though linkage analysis. This family study, conducted from 2001 to 2003, recruited an additional 18 to 25 extended families (a total of about 900 members at least 15 years of age) from each of the field centers, with a total of 3776 subjects from 94 families, of whom 825 were SHS pilot family participants (https://strongheartstudy.org/Research/ResearchDesign.aspx). A genome-wide scan found chromosome linkages for a number of pathological conditions that are more prevalent in the AI/AN population. For example, the scan identified significant linkage of a locus on chromosome 4q35 to weight and BMI for 963 individuals from 58 families from Arizona, Oklahoma, and North and South Dakota [62]. Further analyses of individual study sites revealed the greatest linkage between loci on chromosome 4 to obesity in an AI population from Arizona. Linkage signals for BMI were also observed on chromosomes 5, 7, 8, and 10 in another study [62]. Genome-wide linkage analysis also identified a significant linkage of a locus for the left carotid artery diastolic and systolic lumen diameter. For instance, a linkage of a locus on chromosome 7 at 120 cM for the left carotid artery diastolic and systolic lumen diameter was identified in the Arizona SHFS participants and a linkage of a locus on chromosome 12 at 153 cM for the left carotid artery diastolic and systolic lumen diameter was found in the Oklahoma SHFS participants [63]. Additionally, a linkage for the right carotid artery diastolic and systolic lumen diameter was also detected on chromosome 9 at 154 cM in the Oklahoma participants [63]. Linkages of chromosome 7p19q with pulse pressure, glucose/insulin/obesity factor to chromosome 4, the dyslipidemia factor to chromosome 12, and the blood pressure factor to chromosome 1 were also identified among SHFS participants [64, 65].

In several studies, North et al. have found evidence for the effect of heritability on the prevalence of DM and CVD in the AI/AN population. For instance, significant heritability of diabetes status [66], as well as several CVD risk factor phenotypes like high density lipoprotein cholesterol and diastolic blood pressure, was demonstrated among AIs [61]. In addition, significant heritability for the common carotid artery diastolic diameter, intimal-medial wall thickness, vascular mass, arterial stiffness, and the augmentation index has been identified in the AI population [60].

### 3.2. Risk Factors Contributing to the Increased Incidence of CVD in the AI/AN Population

Evidence suggests that several risk factors, such as tobacco abuse, DM, hypertension, and elevated cholesterol levels, contribute to the increased incidence of CVD in the AI/AN population [11, 35]. Analysis of the chronic heart disease (CHD) outcome data from SHS revealed that age, gender, total cholesterol, low-density lipoprotein (LDL) cholesterol, high-density lipoprotein (HDL) cholesterol, smoking, DM, hypertension, and albuminuria were significant CHD risk factors in this population [35, 67]. Interviews conducted through the BRFSS of AI adults living in the seven Montana reservations showed an alarmingly high level of modifiable CVD risk factors among those with and without DM [68, 69]. Specifically, tobacco use is a big concern in this population with 26.7% of AI/AN adults identifying as smokers compared to only 15.2% of Asian men, 17.3% of Hispanic men, 23.7% of non-Hispanic black men, and 23.9% non-Hispanic white men, after age-adjusted estimates [70]. Similarly, AI/AN women (20.7%) are more likely to be current cigarette smokers than Asian women (5.5%), Hispanic women (9.6%), non-Hispanic black women (17.6%), and non-Hispanic white women (20.9%) [12]. Physical inactivity is another important risk factor that contributes significantly to CHD development. In 2011, only 17.0% of AI/AN adults (age 18 and older) met the 2008 federal guidelines for physical activity [71]. Obesity or being overweight is another risk factor for CVD prevalent in the AI/AN and general population. Only 27.6% of AI/AN individual maintain a healthy weight; a rate much less than whites (36.6%) or Asians (56.7%) [36].

## 4. Prevention of DM and CVD in the AI/AN Population

### 4.1. Control and Management of Risk Factors for DM and CVD

Physical inactivity, obesity, and hypertension are known major risk factors for DM and CVD. Among these, physical inactivity is modifiable and obesity is a controllable risk factor. DM and CVD are preventable by controlling physical activity and obesity. It is well recognized that exercise and weight loss reduce blood pressure, increase insulin sensitivity, prevent or delay the onset of T2D, lower CVD risk, and reduce the risk for heart attack and stroke [72]. Successful management and control of these modifiable risk factors have been the focus for public health, and it has been shown to reduce the risk of DM and CVD as well as delay its progression and complications, including CVD, in the general population [73]. Furthermore, studies have shown the positive impact of lifestyle interventions on preventing DM and CVD risks in the AI/AN population [74]. For instance, early lifestyle interventions that focused on promoting healthy eating and regular exercise were shown to decrease the development of metabolic syndrome in the Southwestern AI population [74]. A targeted tri-weekly exercise regimen, coupled with nutritional counselling, conducted in a community-based cohort study among 65 Zuni Pueblo adolescents resulted in significant improvements in BMI, fat-free mass, total body fat, and fasting lipid profile after six months [74].

### 4.2. Recognition and Integration of Specific Health Issues of Individual AI/AN Communities into Health Promotion Programs

Substantial variations in the prevalence of DM and CVD among the AI/AN population when compared to the general population have been identified. This fact suggests that race, ethnicity, cultural norms, and historic conditions play an important role in the prevalence of DM and CVD. Recognizing these disparities and incorporating the health issues specific to each community into health promotion programs and policies are a critical step in overcoming the disproportionate toll these illnesses take on a population [75]. Food insecurity, defined as uncertain or inadequate access to enough foods for an active healthy life due to shortage of money or resources, has been linked to obesity in children, DM in adults, and poor glucose control in DM patients [76]. Unfortunately, 23% of AI/AN families had incomes below the poverty level in 2010, compared to 16% of the general population. One study has shown that a high level of social integration was significantly correlated with diabetes management behaviors, including monitoring glucose levels and A1C, maintaining a healthy diet, participating in a regular exercise program, and examining feet [73]. In addition, most AI/AN communities live in rural areas sometimes in isolated (e.g., Alaskan villages) living circumstances, which is a huge barrier to healthy eating due to high cost and long commutes for fresh food [77]. Food insecurity was reported in 40% of households in a study conducted in the rural Northern Plains reservation [78]. Therefore, providing both affordable nutrient-rich food choices near AI/AN communities and dietary intervention at the individual level should be included in environmental intervention plans.

### 4.3. The Special Diabetes Program for Indians (SDPI) with DM Prevention

In order to address the diabetes epidemic among the AI/AN population, congress initiated the Special Diabetes Program for Indians (SDPI) in 1997 to provide funds for DM prevention and treatment and to increase access to quality diabetes care with a focus on effective evidence-based intervention strategies. The SDPI diabetes best practices' program focuses on screening and monitoring glycemic control, blood pressure, cardiovascular complications, retinopathy, gum and tooth diseases, depression, tobacco use, nutrition, and physical activity education [79]. The SDPI diabetes prevention and healthy heart program focuses on translating scientific evidence into prevention and reduction of diabetes and CVD risk factors among AI/AN communities. The SDPI diabetes prevention grant program is designed to reduce DM risk in high-risk individuals through a proven lifestyle change intervention, and the SDPI healthy heart (SDPI-HH) program seeks to reduce CVD risk among AI/ANs with T2D [80]. This program provides standardized health care services for cardiovascular risk reduction which includes assessment of blood sugar and blood pressure, as well as encouraging patient exercise and healthy nutrition [81]. The SDPI-HH model engages patients in the health care team and provides a promising framework for understanding barriers to and solutions for improving health care. Finally, it strives to overcome medical mistrust and addresses the specific health problems faced by the AI/AN population [81].

The SDPI has had a positive impact on prevention and control of diabetes among AI/AN populations (presented in the SDPI 2014 Report to Congress). The increase in diabetes prevalence among AI/AN adults has slowed and has not increased in AI youth, suggesting that key diabetic clinical parameters are under control and more importantly, the incidence of end-stage renal disease in people with diabetes is decreasing [82–86].

### 4.4. Improving CVD Knowledge and Health Literacy Levels in the AI/AN Population

Medical mistrust is defined as a patient feeling uncomfortable, fearful, or suspicious in a health care setting and has been found to be significantly higher in AI/AN populations when compared to whites [87]. Health literacy (HL), which is the ability to acquire, process, and comprehend basic health information, may also have significant implications in medical mistrust. Individuals with inadequate HL skills have been shown to have more restricted knowledge of a variety of health conditions and medical services including DM, chronic heart failure (CHF), and hypertension [88]. 48% of AI/AN adults have limited HL skills compared to 36% of U.S. adults. This discrepancy is likely to be the result of limited educational attainment and a high poverty rate in AI/AN populations [67, 89, 90]. One specific area of concern is knowledge about CVD, which has been shown to be restricted in AI/ANs. For instance, AI/AN knowledge of heart attack and stroke symptoms was more limited than that of the general population [91]. Although they understand the risk of obesity and importance of physical inactivity in CVD development, they are unable to tell what blood pressure values would be considered high [91, 92]. The National Heart, Lung, and Blood Institute (NHLBI) developed the Honoring the Gift of Heart Health (HGHH) curriculum to focus on improving the HL level of AI/AN communities. Improvements in HL levels, including heart attack knowledge, stroke, and general CVD knowledge, have been observed in HGHH curriculum participants [92].

In conclusion, the incidence and prevalence of DM and CVD are much higher in the AI/AN population when compared to any other racial or ethnic groups in the U.S. The high risk of developing DM and CVD complications is also disproportionately higher irrespective of age, gender, or geographical location. Multiple risk factors should be considered when establishing prevention programs to decrease the prevalence of obesity, diabetes, and CVD among adults and children in the AI/AN population. Prevention programs should focus on behavioral risk factors and lifestyle changes such as encouraging smoking cessation, eating a healthy diet, and increasing physical activity. In addition, cultural norms, historical conditions, and individual health issues among AI/AN communities should be acknowledged and integrated into all prevention programs.
